# Cardiac rehabilitation engagement and associated factors among heart failure patients: a cross-sectional study

**DOI:** 10.1186/s12872-023-03470-x

**Published:** 2023-09-11

**Authors:** Tianxi Yu, Min Gao, Guozhen Sun, Guendalina Graffigna, Shenxinyu Liu, Jie Wang

**Affiliations:** 1 School of Nursing, Nanjing Medical University, No.140 Han Zhong Road, Gu Lou District, Nanjing City, Jiangsu Province China; 2 Cardiology Department, The First Affiliated Hospital of Nanjing Medical University, No.300 Guang Zhou Road, Gu Lou District, Nanjing City, Jiangsu Province China; 3 Department of Psychology, Università Cattolica del Sacro Cuore, Milan, Italy

**Keywords:** Chronic heart failure, Cardiac rehabilitation, Patient Engagement, Predictive factors

## Abstract

**Background:**

Chronic Heart Failure (CHF) still affects millions of people worldwide despite great advances in therapeutic approaches in the cardiovascular field. Cardiac rehabilitation (CR) is known to improve disease-related symptoms, quality of life and clinical outcomes, yet implementation is suboptimal, a frequently low engagement in rehabilitation programs has been found globally.

**Objective:**

To quantify diverse CR-engaged processes and elucidate associated factors of the various levels of CR engagement in CHF patients.

**Methods:**

Discharged patients admitted from cardiology departments between May 2022 to July 2022 were enrolled by mobile phone text messaging, CHF patients from same department between August 2022 to December 2022 were enrolled by face-to-face. Individuals who met the inclusion criteria filled the questionnaires, including the generalized anxiety disorders scale, patient health questionnaire, cardiac rehabilitation inventory, patient activation measure, Tampa scale for kinesiophobia heart, social frailty, Patient Health Engagement Scale (PHE-s®). We obtained sociodemographic characteristics and clinical data from medical records. Chi-square tests and multivariable logistic regression analyses were performed to examine the factors associated with CR engagement phases.

**Results:**

A total of 684 patients were included in the study. 52.49% patients were in the Adhesion phase. At the multivariate level, compared with the blackout phase process anxiety, monthly income (RMB yuan) equal to or more than 5,000 were the most important factor impacting CHF patients CR engagement. Compared with the Blackout phase, regular exercise or not, severe depression, previous cardiac-related hospitalizations 1 or 2 times, Age influenced patient CR engagement in the Arousal phase. Besides, compared with the Blackout phase, outcome anxiety and activation level were independent factors in the Eudaimonic Project phase.

**Conclusion:**

This study characterized CR engagement, and explored demographic, medical, and psychological factors—with the most important being process anxiety, monthly income, patient activation, severe depression, and previous cardiac-related hospitalizations. The associated factors of CR engagement were not identical among different phases. Our findings suggested that factors could potentially be targeted in clinical practice to identify low CR engagement patients, and strategies implemented to strengthen or overcome these associations to address low CR engagement in CHF patients.

## Introduction

Chronic heart failure (CHF) is a group of complex clinical symptoms caused by abnormal changes in cardiac structure and/or function, resulting in abnormal ventricular systolic contraction and/or diastolic relaxation [1]. At the terminal stage of various cardiovascular diseases, CHF profoundly affects the functional and psychosocial well-being of patients through its dyspnea, diminished endurance, and psychological distress [2]. Moreover, CHF patients are often limited in their daily activities because of repeated recurrence of symptoms as fatigue, dyspnoea and exhaustion [3]. Improving the prognosis of CHF symptoms and the emergence of new drugs such as ARNI(Angiotensin receptor-neprilysin inhibitor) and instruments of non-drug methods such as cardiac implantable electronic devices, mechanical breathing, blood for the clinical application of ultrafiltration, brought new hope to patients with HF. However, therapeutic effect is not significant in patients with middle and late phase and also failed to reduce mortality of the HF group [4]. Thus, CHF has become one of the main diseases endangering human health in the 21st century [5]. Delaying the progression of CHF has become one of the most important topics in the study of Cardiac disease [6, 7].

Cardiac rehabilitation (CR) is a multidisciplinary, guideline-recommended, secondary prevention program that promotes exercise capacity, reduces cardiovascular risk, and improves health-related quality of life for stable heart failure patients with preserved or reduced ejection fraction [8, 9]. The safety and effectiveness of CR have been confirmed after decades of development, and now CR is recommended by international guidelines as an important model of daily management in CHF patients [10, 11]. Although long-term regular engagement is the premise of highly effective benefits, the majority of eligible patients were not involved in the CR changes. According to statistics, about 74 (66.7%) countries meet the threshold of a mean ≥ 12 sessions. With regard to CR dose in alternative settings, home-based programs were offered by 36 (32.4%) countries [12]. Nevertheless, low rates of referrals, barriers to engagement, and difficulty retaining patients are becoming increasingly common issues worldwide. A study demonstrated that only 16.3% of discharged patients engaged in CR from 2007 to 2011 [13, 14]. Even worse, a mere 18.7% of patients with CHF completed the recommended 24 sessions [15]. Given this, it is urgent to identify the influential factors in CR engagement accurately and precisely, and accordingly devise effective interventions.

**Fig. 1:**
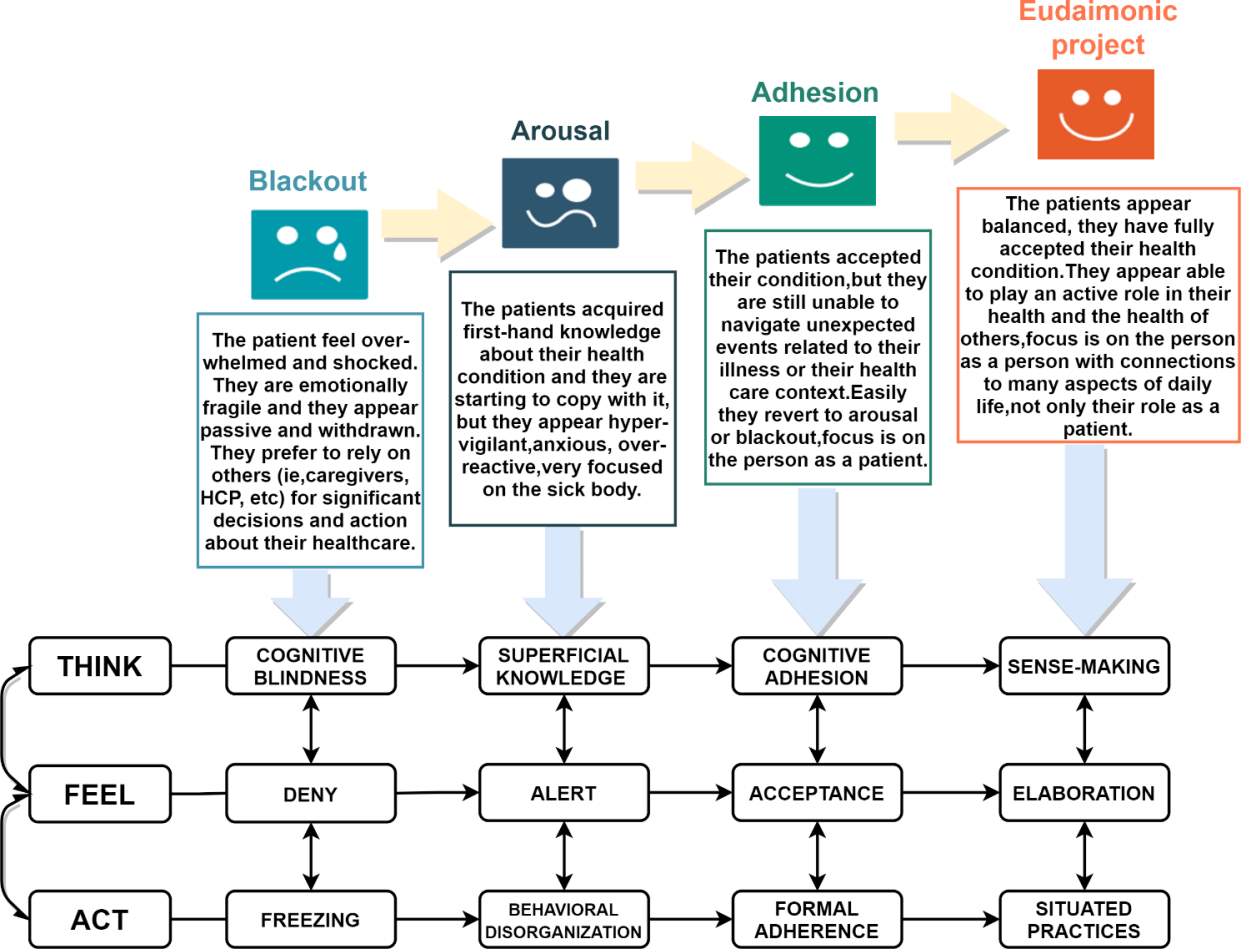
Description of the phases featuring the PHE-Model(Inferred from Ref. [21] and [24]], more details: https://www.researchgate.net/publication/274194432)

The search of available literature revealed that factors associated with poor CR engagement include age, gender, knowledge, self-efficacy, spare time, and social support [16, 17]. Also, distance from home to hospitals may be particularly important in remote areas [18]. In reality, however, CR engagement is a risk-benefit psychological game, with complicacy and variability. The above-mentioned findings did not grasp variability and gave insufficient information in specific time durations, which leads to a lack of targeted intervention approaches. Under the driving of the “Healthy China 2030” strategy [19], we witnessed a paradigm shift in clinical practice from a disease-centered to a patient-centered [20]. In this context, patients themselves are garnering more research and clinical attention as the key resources in health management. With the emergence of a new concept of engagement, we found tools to better explain the complex psychological changes involved in cardiac rehabilitation. The concept of “Engagement” emphasizes the equality between medical staff and patients and believes that patients real disease experiences and specific needs are key to guiding the direction of medical action and addressing priorities. The model we use in this research is Patient Health Engagement Model (PHE-Model) established on the concept above, which could take patients themselves into account and evaluate the complex and dynamic psychological nature of the patient engagement experience accurately [21]. The Chinese version of the PHE-s® scale has only five items and is easy to answer due to its shortness and could easily judge the position at which the patient is currently at (i.e., Blackout, Arousal, Adhesion, Eudaimonic Project, see Fig. 1 for specific meaning) [22]. In addition, we have applied the PHE-Model to CHF patients before using it.

In China, given its large HF population and poor accessibility, home-based CR is becoming strongly recommended for patients in the stable stage [14, 15]. Therefore, it is particularly important to seek relevant factors of home-based CR engagement. Indeed, the paucity of evidence in such populations may have particular implications, because the number of hospitals performing cardiac rehabilitation was 13.2 per 100 million population [23], and most CHF patients may not able to available CR services currently.

However, there are inadequate findings on CR engagement and its related factors in patients with CHF, particularly the psychometric properties in home-based CR.

The aim of this study was to explore factors that affect CR engagement in different phases in CHF patients to identify potential targets for future interventions to improve CR engagement. The hypothesis of the study is as follows:

### Hypothesis 1

For CR engagement, most CHF patients will be concentrated in the Arousal and adhesion phases.

### Hypothesis 2

The associated factors of CR engagement will not identical among different phases in CHF patients.

## Methods

### Design and sample

A cross-sectional survey was conducted on participants recruited by convenience sampling in six cardiology units of the First Affiliated Hospital of Nanjing Medical University from May 1 2022 to November 1 2022 by structured questionnaires. All research subjects have signed informed consent forms prior to participating in the study. The procedure was in accordance with the declaration of Helsinki. The patients met the following inclusion criteria: (1) a definite diagnosis of heart failure by a cardiologist; (2) age ≥ 18 years. Excluded patients: (1) With a physical impairment that would seriously impair their physical mobility (e.g., physical disabilities); (2) Acute phase of Heart Failure; and (3) had severe mental or cognitive impairments documented in medical records, or without the ability to complete the survey. The study was approved by the Ethics Committee of the First Affiliated Hospital of Nanjing Medical University Ethics Committee.

### Procedure and data collection

Prior to this survey, we sent personal invitation letters to patients and explained the purpose and processes of this study to all potential participants. Discharged participants were recruited by telephone and in hospital patients’ were face-to-face. The questionnaires were completed by telephone inquiry or the internet through sending a link to their mobile phones. We obtained every participant’s demographic characteristics including age, level of education, marital status and so on. We also collected the disease-related characteristics of every participant. Clinical data and part of general information on patients with heart failure were obtained through reviews of medical records. Cardiac Rehabilitation inventory, cardiac anxiety, depression, social frailty and patient activation were assessed using self-reported questionnaires.

### Measure

#### Sample characteristics

While all of the subjects filled the questionnaire (age, gender, smoking and drinking status, monthly income, disease course, level of education, NYHA class, left ventricular ejection fraction [LVEF, set as reduced LVEF (≤ 40%), mildly reduced LVEF (41–49%), and preserved LVEF (≥ 50%)], NT-pro-BNP, comorbidities, and history of admission for cardiac disease, etc.) In addition, patients completed the Cardiac Rehabilitation Inventory scale, the Generalized Anxiety Disorders Scale, the Patient Health Questionnaire, the Social Frailty scale, and the Patient Activation Measure.

#### The generalized anxiety disorders scale

For assessing anxiety, the 7-item Generalized Anxiety Disorders Scale (GAD-7) [25], validated for use in Chinese [26], was applied, which is recommended and reliable tool for CHF patients to measure generalized anxiety disorder was used to assess patients’ anxiety. The answers are given in a scale from 0 to 3, respective to the frequency of the symptoms (0 = not at all, 1 = several days, 2 = more than half of the days, and 3 = nearly every day). The total score ranges from 0 to 21, where a higher score means more severe anxious symptomatology. Scores of 5, 7, 11, and 18 represent thresholds demarcating the lower limits of tendency, mild, moderate, and severe anxiety, respectively. The Cronbach’s α = 0.898 for GAD-7 [27, 28].

#### The patient health questionnaire

The Patient Health Questionnaire (PHQ-9) was used to measure the severity of depressive symptoms in patients with heart failure. There were nine items (e.g., ‘Little interest or pleasure in doing things) [29]. Participants were asked to rate how often they had been bothered by any of the problems over the previous 2 weeks on a 0–3 point scale, where 0 = Not at all to 3 = Nearly every day. The total score is the sum of the items, and a higher score indicates a more serious degree of depression. Scores of 5, 8, 15, and 22 represent thresholds demarcating the lower limits of tendency, mild, moderate, and severe depression, respectively. The PHQ-9 is valid and reliable and has been widely used in studies with cardiac patients. The Cronbach’s α was 0.93 [30].

#### Cardiac rehabilitation inventory

We used Cardiac Rehabilitation Inventory (CRI) to access patients’ rehabilitation needs [31]. The survey tool is based on the Theory of Planned Behaviour (TPB) and the Transtheoretical Model, which includes three dimensions (Outcome anxiety, Process anxiety, and Autonomy). A 5-point Likert response set was used for each item: strongly agree, agree, undecided, disagree, and strongly disagree. Each response was given a score ranging from 0 to 4, with positive items receiving a score of 4 for strongly agree responses and 0 for strongly disagree responses. In addition, a score on the Autonomy of ≤ 15 is defined as low autonomy; For the dimension “Process anxiety”, If the score is ≥ 19, it indicates that the patient may have process anxiety; In Outcome anxiety, a score of ≥ 10 is highly suggestive of a problem with anxiety. The scale’s Cronbach’s α coefficient in this study was 0.868 [32].

#### Patient activation measure

Patient activation was measured by the simplified Chinese version of the 13-item Patient Activation Measure (PAM-13®) [33, 34]. PAM® results range from 0 to 100 (higher scores indicating higher activation) and are converted to 4 levels and labeled as follows: (1) Disengaged and overwhelmed, (2) Becoming aware, but still struggling, (3) Taking action, and (4) Maintaining behaviors and pushing further. The questionnaire is composed of four dimensions: cognition, skill, action, and belief. The questionnaire has been validated for multiple language editions with all adequate clinimetric properties. Cronbach’s α coefficient for the total scale is 0.82 in this study [35]. Besides, we have obtained permission to use it.

### Tampa scale for kinesiophobia heart

The TSK-Heart was used to measure fear of movement in patients with heart failure [36]. It has shown good reliability and validity in Chinese cardiovascular patients [37]. The scale consists of 15 items and is separated into four sub-dimension [Perceived danger for heart problem (Danger), Avoidance of exercise (Avoidance), Fear of injury (Fear), Dysfunctional self (Dysfunction)]. Each item is rated from ‘strongly disagree’ (score = 1) to ‘strongly agree’ (score = 4), while items 6 and 15 are scored in reverse [38]. The total score ranges from 15 to 60, with higher scores indicating higher levels of fear of movement. The Cronbach’s α for the TSK-Heart was 0.76 in the current study.

### Social frailty

Patients’ social frailty was assessed using simple 5-item questions regarding living alone, going out less frequently compared with the prior year, visiting friends sometimes, feeling helpful to friends or family, and talking with someone every day, developed based on previous studies to explore the relationship between its social frailty determinants and specific functional decline [39]. Social frailty is defined as meeting at least 2 criteria and prefrailty meeting 1 criterion. To test the suitability of the scale in heart failure patients, we performed the internal consistency reliability (Cronbach’s α = 0.769) and confirmatory factor analysis (CFA) on the scale.

### Patient health engagement scale (PHE-s®)

Finally, the Chinese version Patient Health Engagement Scale (CPHE-s**®**) was used to assess the patient’s psychological readiness to take an active role in their CR [22]. This scale was developed according to the Patient Health Engagement Model which features four “positions” along a continuum of patient engagement (i.e., Blackout, Arousal, Adhesion, Eudaimonic Project) [21]. This scale can assess the level of patients’ engagement, and it consists of five short items. Answers are collected on a 7-point scale (lower scores meaning a patient engagement level closer to the “Blackout” position, higher scores indicating a patient engagement level closer to “Eudaimonic project”). The peculiarity of this scale is that it allows not only to assess the patient’s attitude toward their health condition but also to forecast the patient’s risk for disengagement in disease management [38]. Prior to data collection, we performed pre-tested, and satisfactory results were obtained.

**Table 1:** Research tools

Variables	Tool
Independent Variables	
Negative emotions	The Generalized Anxiety Disorders Scale
	The Patient Health Questionnaire
CR Needs	Cardiac Rehabilitation Inventory
Kinesiophobia	Tampa Scale for Kinesiophobia Heart
Social resource satisfaction	Social Frailty
Dependent variable	
CR engagement	Patient Health engagement scale (PHE-s®)

### Data analysis

In terms of descriptive statistics, categorical variables were presented as frequencies and percentages, and continuous variables were presented as means and standard deviations. In dissimilarity tests, categorical data were tested using the chi-square test and Fisher’s exact test. Analogously, the correlation was tested with the Spearman correlation test in observed variables because the CR engagement was in Multiple classification distribution. After confirming the eligibility of the assumptions for disordered multiclass logistic regression, multiclass logistic regression analysis showed that the factors associated with engagement were assessed while controlling for confounding variables (Gender and Age). The variables included in the multivariable logistic regression analysis were categorized variables. The responses were not included in the analysis when more than 95% of individuals had the same response to the categorical independent variables. Findings were considered statistically significant when a two-tailed P value was < 0.05 in SPSS 27.0 (IBM Corp, Armonk, New York).

## Results

### Demographics of the study sample

A total of 684 patients were included in the study with 243(35.5%) inpatients and 441(64.5%) outpatients. Among them, more men (70.9%) than women (29.1%). The average age of the 684 patients was 58.34 (SD 13.696), and 32.3% (*n* = 221) were over 65 years old. Less than half of the patients (47.8%, *n* = 327) were with NYHA class II, and 47.2% of patients (*n* = 323) had a history of heart disease for 1 to 5 years. Table 1 shows the distribution of the socio-demographical and clinical characteristics of the sample included in the study.

### Comparisons of the CR engagement phases for CHF patients

Table 2 compares the characteristics of patients in different PHE-s® phases. Comparison analyses by demographic characteristics showed that males were more likely to engage in CR than female patients (*x*^*2*^ = 8.081, *P* = 0.044). Besides, age, full-time education, monthly income, and marital status also affected patients’ CR engagement phases. At the same time, we also got patients’ living habits data, and find that high engagement in patients who have exercise habits before and low PHE-s® phases in drinking patients. In terms of the comparison of clinical characteristics, patients with a differ Cardiac-related hospitalizations and LVEF levels imply different degrees of CR engagement. CHF patients who dependent on pacemakers had different CR engagement than those who do not.

**Table 2:** Sample characteristics

	Blackout n (%) N = 37	Arousal n (%) N = 162	Adhesion n (%) N = 359	Eudaimonic Project n (%) N = 126	Chi-Square/Fisher	*P*
Age (year)					14.601^a)^	0.024
18 ~ 44.9	3(8.11)	34(20.99)	35(9.75)	13(10.32)		
45 ~ 65	21(56.76)	81(50.00)	207(57.66)	70(55.56)		
>65	13(35.14)	47(29.01)	117(32.59)	43(34.13)		
Gender					8.081^a)^	0.044
Male	22 (4.5)	105(21.6)	261 (53.8)	97 (20.0)		
Female	15 (7.5)	57(28.6)	98 (49.2)	29 (14.6)		
Full-time Education					16.295^a)^	0.012
Did not graduate high school	21 (9.6)	51(23.4)	105(48.2)	41 (18.8)		
High school graduate/ GED	6 (3.2)	54 (28.4)	101(53.2)	29 (15.3)		
College Graduate	10 (3.6)	57(20.7)	153 (55.4)	56 (20.3)		
Monthly income					17.189^a)^	0.009
<3000 yuan	19 (10.5)	47(26.0)	91 (50.3)	24 (13.3)		
3000 ~ 5000 yuan	9 (4.5)	48 (24.2)	102 (51.5)	39 (19.7)		
>5000 yuan	9 (3.0)	67 (22.0)	166 (54.4)	63 (20.7)		
Marital status					8.775^b)^	0.032
Married/cohabiting	33 (5.4)	137(22.3)	323 (52.7)	120 (19.6)		
Not in a relationship	4 (5.6)	25(35.2)	36 (50.7)	6 (8.5)		
Living habits						
Drinking (yes)	4 (2.8)	25 (17.2)	84 (57.9)	32 (22.1)	7.984^a)^	0.046
Smoking (yes)	6 (5.0)	27 (22.3)	66 (54.5)	22 (18.2)	0.295^a)^	0.961
exercise habits (yes)	23 (5.5)	81(19.5)	224 (53.8)	88 (21.2)	12.664^a)^	0.005
low-salt diet (yes)	29 (5.5)	117(22.2)	283 (53.8)	97 (18.4)	5.413^a)^	0.492
NYHA					4.463^b)^	0.870
I	14 (5.1)	72 (26.1)	140 (50.7)	50 (18.1)		
II	20 (6.1)	70 (21.4)	176 (53.8)	61 (18.7)		
III	2 (2.8)	18 (25.0)	39 (54.2)	13 (18.1)		
IV	1 (11.1)	2 (22.2)	4 (44.4)	2 (22.2)		
LVEF					15.482^b)^	0.012
< 40%	2 (4.2)	3 (6.3)	33 (70.0)	10 (20.8)		
40–49.9%	0 (0.0)	12 (34.3)	15 (45.5)	6 (17.1)		
≥ 50%	35 (5.8)	147(24.5)	309 (51.4)	110 (18.3)		
HF duration (year)					12.463^a)^	0.052
<1	6 (4.2)	40 (28.0)	65 (45.5)	32 (22.4)		
1 ~ 5	22 (6.8)	81 (25.1)	161 (49.8)	59 (18.3)		
>5	9 (4.1)	41 (18.8)	133 (61.0)	35 (16.1)		
Cardiac-related hospitalizations					32.887^a)^	<0.001
0	13 (6.5)	43 (21.6)	86 (43.2)	57 (28.6)		
1 ~ 2	14 (4.2)	85 (25.6)	182 (54.8)	51 (15.4)		
2 ~ 5	4 (3.6)	27 (24.3)	69 (62.2)	11 (9.9)		
>5	6 (14.3)	7 (16.7)	22 (52.4)	7 (16.7)		
have a pacemaker (yes)	8 (11.9)	6 (9.0)	37 (55.2)	16 (23.9)	21.828^b)^	<0.001
Hypertension (yes)	11 (4.1)	69 (25.7)	142 (53.0)	46 (17.2)	2.577^a)^	0.462
Coronary heart disease (yes)	13 (4.2)	67 (21.8)	170 (55.4)	57 (18.6)	3.127^a)^	0.372
Diabetes (yes)	7 (4.4)	33 (20.6)	90 (56.3)	30 (18.8)	1.815^a)^	0.612

NYHA: New York Heart Association; LVEF: Left Ventricular Ejection Fractions; have exercise habits: Equal to or more than 3 times weekly, over 30 min each time. a: multiset chi-square testing, b: fisher

### Description and correlation coefficients of study variable

As shown in Table 2, The most frequent PHE-s**®** phase was Adhesion (*n* = 358), comprising 52.49% of the total patients, Arousal comprising 23.68%(*n* = 162), and the Eudaimonic Project comprising 18.42%(*n* = 126). In Table 3, the median GAD scores were 3 (1, 8) in the respondents, and PHQ scores were 4 (1, 7), of those, 22.08% of the patients scored ≥ 7 points for anxiety and 19.88% of the patients scored ≥ 8 points for depression, which is an indication of significant psychological comorbidity. The average score for CRI Autonomy was 24.21(4.33), which means that patients may have high autonomy. But the other two dimensions were not ideal, with 20.66(4.62) in Process Anxiety and 12.43(3.71) in Outcome Anxiety. The median score for PAM was 53.2(53.2, 65.5). From the rank rating, most patients located in the 3 level (*n* = 280, 40.9%), which means that patients received the active treatment gradually, but still lacks the confidence and skills to support their actions. For TSK-heart, the average score was 34.21(11.19). Regarding dimensions, the avoidance of exercise (avoidance) dimension had the highest score (Average,3.09; SD: 1.15), followed by dysfunctional self (Dysfunction), and perceived danger for heart problems (Danger). In addition, 266(38.89%) CHF patients have social frailty.

**Table 3:** Descriptive statistics

Variables	Descriptive statistics
PHE-s® phase	3(1,4) ^a)^
PHQ9	4(1,7) ^b)^
GAD7	3(1,8) ^b)^
CRI-1	24.21(4.33) ^c)^
CRI-2	20.66(4.62) ^c)^
CRI-3	12.43(3.71) ^c)^
PAM13®	53.2(53.2,65.5) ^b)^
TSK-heart	34.21(11.19) ^c)^
SF	2(1,2) ^b)^

^(**a**)^ M (Min, Max); ^(**b**)^ M (M_25_, M_75_); ^(**c**)^ Mean (SD);

In Table 4, Spearman correlation analyses showed that GAD scores (*r*=-0.423, P < 0.001), PHQ scores (r=-0.353, *P* < 0.001), and social frailty scores (*r*=-0.269, *P* < 0.001) were negatively correlated with the PHE-s® phase, whereas the PAM® scores (*r* = 0.234, *P* < 0.001) were positively correlated with the PHE-s® phase in heart failure patients. Moreover, the PHE-s® phase was positively correlated with the CRI Autonomy (*r* = 0.085, *P* < 0.05), and negatively correlated with the CRI Process Anxiety (*r*=–0.337, *P* < 0.001). However, there was no correlation between the PHE-s® phase and TSK-heart (*r* = 0.072, *P* > 0.05), possibly owing to a comprehensive and personalized CR being more than controlled exercise training.

**Table 4:** Correlation coefficients of the score for patient engagement with scores for other continuous variables in patients with heart failure (*n* = 684)

Variables	PHE-s® phase	PHQ9	GAD7	CRI-1	CRI-2	CRI-3	PAM13®	TSK	SF
PHE-s® phase	1								
PHQ9	-0.353**	1							
GAD7	-0.423**	0.738**	1						
CRI-1	0.085*	-0.112*	-0.134**	1					
CRI-2	-0.337**	0.433**	0.449**	-0.015	1				
CRI-3	-0.055	0.209**	0.224**	-0.126**	0.437**	1			
PAM13®	0.234**	-0.242**	-0.218**	0.353**	-0.163**	-0.219**	1		
TSK-heart	0.072	-0.491**	-0.368**	0.314**	-0.175**	-0.175**	0.087*	1	
SF	-0.269**	0.379**	0.351**	-0.215**	0.221**	0.106*	-0.188**	-0.340**	1

*P<0.05;**P<0.001; CRI-1: Autonomy; CRI-2: Process Anxiety; CRI-3: Outcome Anxiety.

### The multinomial logistic regression analysis of patient health engagement

In the multinomial logistic regression model, the PHE-s® phase for patients’ CR engagement was entered as the dependent variable, factors with univariate significance at the 5% level (*P*<0.05) were entered as the independent variable, such as full-time Education(*P* = 0.012). In Table 5, we assigned values to independent variables. Adjusted covariate by gender, the aforementioned variables were entered into the multinomial logistics regression model that was created, see Table 6. The logistic regression analysis recorded a significant Omnibus test for the model (significance < 0.001). The Pseudo R^2^ statistic indicated that the model, as a whole explained between 38.1% (Cox & Snell *R*^*2*^ = 0.434) and 42.3% (Nagelkerke *R*^*2*^ = 0.423) of the variance in CR engagement. We used the first category as a reference to ensure the directionality of variables and ease of understanding the results. The results were obtained as follows: Relative to the CR Engagement Blackout phase, the process anxiety was a significant associated factor of other CR engagement phases (Arousal: OR 0.829, 95%CI: 0.73 ~ 0.94; Adhesion: OR 0.725, 95%CI: 0.64 ~ 0.82; Eudaimonic Project: OR 0.674, 95%CI: 0.59 ~ 0.77). Similarly, individuals with a monthly income (RMB yuan) equal to or more than 5,000 had a greater positive impact on the patients’ CR engagement phase compared to the first engagement phase (Arousal: OR 6.342, 95%CI: 1.30 ~ 31.01; Adhesion: OR 5.226, 95%CI: 1.09 ~ 24.96; Eudaimonic Project: OR 6.658, 95%CI 1.26 ~ 34.76). In addition, a monthly income between 3000 and 5000 was more likely to engage in the Eudaimonic Project phase (OR 4.40, 95% CI: 1.20 ~ 16.19) when compared with those less than 3000. Furthermore, in the Arousal phase, versus the Blackout phase, regular exercise or not (OR 3.29, 95% CI: 1.19 ~ 9.10), Severe Depression (OR 0.019, 95% CI: 0.00 ~ 0.813), previous cardiac-related hospitalizations 1 or 2 times (OR 3.75, 95% CI: 1.19 ~ 11.86), Age (OR 0.958, 95% CI: 0.92 ~ 0.998) influenced patient CR engagement. In the Adhesion phase, patients activate in level 3 (OR 5.017, 95% CI: 1.05 ~ 23.89), Severe Depression (OR 0.013, 95% CI: 0.00 ~ 0.53), Previous Cardiac-Related Hospitalizations 1 or 2 times (OR 3.33, 95% CI: 1.08 ~ 10.24) were independent factors for patient CR engagement in contrast to the Blackout phase. Moreover, the results of the analysis indicated that compared to the Blackout phase, Outcome Anxiety (OR 1.269, 95% CI: 1.11 ~ 1.46) and activation level (level 2: OR 9.357, 95% CI: 1.44 ~ 60.68; level 3: OR 29.96, 95% CI: 3.67 ~ 244.92; level 4: OR 29.71, 95% CI: 3.62 ~ 243.61) were independent factors predicting high CR engagement in the Eudaimonic Project phase.

**Table 5:** Assignment of the independent variable

Independent variable	Assignment
Gender	Male = 1; Female = 2
Full-time Education	Did not graduate high school = 1; High school graduate/ GED = 2; College Graduate = 3
Monthly Income	<3000 yuan = 1; 3000 ~ 5000 yuan = 2; >5000 yuan = 3
Marital status	Married/cohabiting = 1; Not in a relationship = 2
Drinking	Yes = 1; No = 2
Exercise habits	Yes = 1; No = 2
LVEF	< 40%=1; 40 ~ 49.9%=2; ≥50%=3
Cardiac-related hospitalizations	No = 1; 1 ~ 2times = 2; 2 ~ 5times = 3; >5times = 4
have a pacemaker	Yes = 1; No = 2
Depression	No = 1; Tendency = 2; Mild = 3; Moderate = 4; Severe = 5
Anxiety	No = 1; Tendency = 2; Mild = 3; Moderate = 4; Severe = 6
PAM-13	Disengaged and overwhelmed = 1; Becoming aware, but still struggling = 2; Taking action = 3; Maintaining behaviors and pushing further = 4
SF	No = 1; prefrailty = 2; Social Frailty = 3
CRI-Autonomy	Continuous variable
CRI-Process Anxiety	Continuous variable
CRI-Outcome Anxiety	Continuous variable
Age	Continuous variable

**Table 6:** Multinomial logistic regression model examining factors of CHF patients’ engagement

								95%CI Exp(B)	
PHE phase		B	Standard Error	*z* value	Wald χ2	Sig.	Odds Ratio Exp(B)	Lower	Upper
2	Intercept	0.791	2.654	0.298	0.089	0.766	2.205	0.012	400.61
	CRI- Process Anxiety	-0.187	0.062	-2.994	8.963	0.003	0.829	0.734	0.937
	Age	-0.042	0.02	-2.056	4.226	0.04	0.959	0.921	0.998
	[Regular Exercise = 1]	1.19	0.52	2.291	5.248	0.022	3.288	1.188	9.101
	[Monthly Income = 3]	1.847	0.81	2.281	5.204	0.023	6.342	1.297	31.009
	[Depression = 5]	-3.948	1.908	-2.069	4.279	0.039	0.019	0	0.813
	[Cardiac-related hospitalizations = 2]	1.322	0.587	2.25	5.063	0.024	3.75	1.186	11.858
3	Intercept	5.69	2.546	2.235	4.995	0.025	296.001	2.014	43512.068
	CRI- Process Anxiety	-0.321	0.063	-5.133	26.346	<0.001	0.725	0.642	0.820
	[PAM-level = 3]	1.613	0.796	2.026	4.103	0.043	5.017	1.054	23.887
	[Monthly Income = 3]	1.654	0.798	2.073	4.296	0.038	5.226	1.094	24.959
	[Depression = 5]	-4.33	1.887	-2.294	5.262	0.022	0.013	0.000	0.532
	[Cardiac-related hospitalizations = 2]	1.202	0.574	2.096	4.392	0.036	3.328	1.081	10.244
4	Intercept	4.225	2.806	1.506	2.267	0.132	68.384	0.279	16736.106
	CRI- Process Anxiety	-0.394	0.068	-5.817	33.838	0	0.674	0.590	0.770
	CRI- Outcome Anxiety	0.239	0.071	3.363	11.307	0.001	1.269	1.105	1.459
	[Monthly Income = 3]	1.896	0.843	2.249	5.056	0.025	6.658	1.275	34.755
	[Monthly Income = 2]	1.481	0.665	2.229	4.967	0.026	4.399	1.196	16.186
	[PAM-level = 4]	3.392	1.073	3.159	9.982	0.002	29.713	3.624	243.609
	[PAM-level = 3]	3.4	1.072	3.171	10.058	0.002	29.959	3.665	244.919
	[PAM-level = 2]	2.236	0.954	2.344	5.496	0.019	9.357	1.443	60.677

Cox & Snell *R*^2^: 0.381; Nagelkerke *R*^2^: 0.423; Compared to ‘Blackout’ phase

## Discussion

Before the initiation of CR engagement behavior, it is necessary to undergo a complex and continuously dynamic multi-dimensional psychosocial change process, individual’s overall perception of their own health status and CR, emotions and feelings experienced with heart disease, perceptual behavioral control ability greatly affects the formation and maintenance of their behavior. In this study, we used PHE-Model to conduct a cross-sectional study to explore the associated factors for CR engagement in CHF patients’ psychological level. The present study found that 359(52.49%) patients in the Adhesion phase may accept their CHF condition, but was still unable to navigate unexpected events related to their illness or their healthcare context. They often focus on the person as a patient. 162(23.68%) patients in the Arousal phase had acquired first-hand knowledge about their health conditions and began to understand and learn, but they appear hypervigilant, anxious, over-reactive and focused on the sick body. Alternatively, 126(18.42%) patients in the Eudaimonic Project phase had fully accepted their health condition. They appear able to play an active role in their health and can fully utilize various resources to engage and adhere to cardiac rehabilitation. Moreover, 37 (5.41%) patients in the Blackout phase feel overwhelmed and shocked by mood disorders, behavioral rejection, and cognitive deficits. They immersion in the experience of disease and do not have access to effective coping strategies [39, 40]. Surprisingly, in our survey of 684 individuals, we found CHF patients’ engagement was better compared to previous studies. This might be due to the patient’s social desirability effect. In order to create a positive impression, patients tend to replace real information or intention with false information or intention in the survey to conform to social expectations [41].

The logistic regression results showed that the Kinesiophobia Heart was not significantly predictive of CR engagement for both inpatient and outpatient groups, even though there were significant correlations. But in fact, previous findings confirmed the universality of Kinesiophobia, which is a barrier to adherence to physical activity recommendations in elderly patients with various cardiac diseases [42]. We consider exercise training is the core component of a CR program, in deed, CR does not merely consist of exercise. This may have contributed to the negative result of CR engagement. Besides, a higher proportion of patients with mild or moderate heart failure might also account for this.

Although we found that these predictive factors covary and complexity, patients with process anxiety and more than 5000 monthly income were significant in all CR engagement phases compared to blackout patients. Like some other studies, individuals of higher economic status were more aware than those of lower economic status of relevant public health knowledge and were thus more likely to engage CR, in part because they had more psychological resources [43, 44]. Dispositions of apprehension that are specifically associated with the CR process itself may have led to poor CR engagement. This CRI subscale process anxiety reflects some of the barriers that have been identified by others, such as low self-efficacy [45], worries about exercising in front of others [46, 47]. In our survey, CRI Process Anxiety could influence patients’ engagement in whole rehabilitation, reminding us that examples of how this might be changed include varying the induction process, refining age-appropriate activities, or using peer buddies or CR mentors. These or other similar methods might also help increase feelings of autonomy, which, somewhat unsurprisingly, were found to be lowest among those 45 to 60 years. High levels of psychological and financial stress in middle-aged may lead to low autonomy.

Furthermore, severe depression is one of the influencing factors in the two middle phases. Studies have revealed that patients hospitalized for HF experienced a high burden of symptoms, particularly depression [48]. As is well known, mind-body interaction happens at any time. It is certainly possible that poor emotion regulation, which can contribute to mood disturbances, precedes poor sleep or other symptoms that may lead to physical discomfort. In this context, patients are more likely occur physical discomfort. However, the analyzed samples were not perfectly distributed across the entire phases, so we were not able to determine the effect between depression and the Eudaimonic Project phase.

In the Arousal phase, patients with regular exercise habits exhibit less engagement CR than patients with irregular habits. On the one hand, everyone’s idea of regular exercise is different, perhaps physical activity such as walking after meals is also defined as regular exercise. And on the other hand, as a special type of consumer, patients tend to be creatures of habit, unwilling to break existing habits instead of trying new things [49]. In fact, individuals repeatedly performing a behavior in a stable context can develop habits that coincide with their final goal [50, 51]. In view of appeals, it is important to help patients form regular exercise habits after the occurrence of motor behavior. We propose the adoption of these methods for CHF patients to be feasible given the similarities between behavioral requirements, which specifically include being able to safely engage in exercise in an outpatient environment [52]. Unsurprisingly, Age was the negative factor of CR engagement in the Arousal phase, this could be due to the varying degrees of frailty with advancing age [53]. It has been reported that frailty represents one of the major challenges for cardiac rehabilitation community and may contribute to poor functional status and worse clinical outcomes [54]. Frailty was shown to occur frequently in patients with heart failure, with the prevalence ranging from 15 to 74%, depending on the studied population and the method of assessment [55]. In parallel, the older the age was, the higher the complication ratio was, which was the obstacle factor for CR engagement [56].

Meanwhile, previous cardiac-related hospitalizations 1 or 2 times were the positive factor in the middle engagement phase. Hospitalizations are common after HF diagnosis, with 83% of patients hospitalized at least once and 43% hospitalized at least four times [57]. CR is recognized as integral to comprehensive care and the best medicine for HF patients [58]. To minimize the increasing medical costs among patients with HF, further efforts are required to shorten the hospitalization period and prevent HF recurrence. At this time point, CHF patients may be in a time of thirst and drawing for healthy knowledge and show higher psychological engagement [59]. This result suggests that providing patients with health education tailored to their needs and make patients a holistic understanding of the disease is necessary for the Arousal phase. We must also support them in managing their illnesses and coping with illness, thus preventing care dropouts. Samely, patients in the Adhesion phase need to be assisted by medical staff who can help them maintain correct health behavior even in stressful or atypical situations. At this phase, CHF patients may awareness of their role identity, not only as patients but also as persons who are active partners in the medical course.

Interestingly, Dominic defined outcome anxiety in the context that patients focus on beliefs about experiencing negative outcomes either as a direct or an indirect consequence of CR as apprehensive feelings, thoughts, or dispositions [31]. With the data currently available, we may not definitively explain this discrepancy that outcome anxiety was a positive factor in our study.

Of significant mention was the importance of patient activation in patient CR engagement. This study demonstrated that patients’ CR engagement was enhanced as long as patient activation level improved in the Eudaimonic Project phase. Although patient activation may focus on the conative dimension of the behavior of the patient, establishing the intention of CR engagement is a cornerstone of behavior, as well as a top priority [60]. This strategy perhaps provides an efficient path toward improved CR engagement in those CHF patients who have no behavioral intention.

### Strengths and limitations of the study

This is the first study, to our knowledge, to adapt the PHE-Model for application with CR in a sample of patients with CHF. Moreover, we quantified the CR engagement situation and examined the factors predicting different CR engagement phases. This study investigated CHF patients in-hospital as well as after discharge. Obviously, our study would greatly benefit from a wider sampling of the studied population.

There were some limitations to the current study. First, we have adjusted gender in the analyses, but the potential sources of heterogeneity are still unknown. Second, due to the cross-sectional design, longitudinal studies are needed to examine causal or bi-directional relationships and determine how these relationships change over time. Third, although our assessment instruments had good reliability and validity, the self-report form may inevitably lead to reporting bias, for example, the prevalence of depressive symptoms may be higher than that of depression diagnosed clinically.

## Conclusion

It is worth noting that cardiac rehabilitation is still in the early phase in China, and actually most hospitals are not able to provide cardiac rehabilitation services currently due to a variety of reasons. The active home-based rehabilitation of patients is particularly important. In these scenarios, the engagement of patients with CHF who need offering CR to reduce the risk of further cardiac events and to improve patients’ health and quality of life should be improved. Our study suggested that the associated factors of CR engagement were not the same among different phases, to summarize, this strongly suggests a significant role for the PHE-Model and engagement phases and affirms our work. These are important findings, suggesting factors that could potentially be targeted in clinical practice to identify low CR engagement patients, and various CR engagement strategies implemented accordingly to strengthen or overcome these associations to address low CR engagement in individuals.

## Data Availability

The datasets generated and/or analysed during the current study are not publicly available due patient privacy and scales copyright but are available from the corresponding author on reasonable request.
